# Population-size history inferences from the coho salmon (*Oncorhynchus kisutch*) genome

**DOI:** 10.1093/g3journal/jkad033

**Published:** 2023-02-10

**Authors:** Eric B Rondeau, Kris A Christensen, David R Minkley, Jong S Leong, Michelle T T Chan, Cody A Despins, Anita Mueller, Dionne Sakhrani, Carlo A Biagi, Quentin Rougemont, Eric Normandeau, Steven J M Jones, Robert H Devlin, Ruth E Withler, Terry D Beacham, Kerry A Naish, José M Yáñez, Roberto Neira, Louis Bernatchez, William S Davidson, Ben F Koop

**Affiliations:** Department of Biology, Centre for Biomedical Research, University of Victoria, Victoria, BC, V8W 2Y2, Canada; Fisheries and Oceans Canada, 4160 Marine Drive, West Vancouver, BC, V7V 1N6, Canada; Fisheries and Oceans Canada, Pacific Biological Station, 3190 Hammond Bay Road, Nanaimo, BC, V9T 6N7, Canada; Department of Biology, Centre for Biomedical Research, University of Victoria, Victoria, BC, V8W 2Y2, Canada; Fisheries and Oceans Canada, 4160 Marine Drive, West Vancouver, BC, V7V 1N6, Canada; Department of Biology, Centre for Biomedical Research, University of Victoria, Victoria, BC, V8W 2Y2, Canada; Fisheries and Oceans Canada, 4160 Marine Drive, West Vancouver, BC, V7V 1N6, Canada; Department of Biology, Centre for Biomedical Research, University of Victoria, Victoria, BC, V8W 2Y2, Canada; Fisheries and Oceans Canada, 4160 Marine Drive, West Vancouver, BC, V7V 1N6, Canada; Department of Molecular Biology and Biochemistry, Simon Fraser University, Burnaby, BC, V5A 1S6, Canada; Department of Biology, Centre for Biomedical Research, University of Victoria, Victoria, BC, V8W 2Y2, Canada; Department of Biology, Centre for Biomedical Research, University of Victoria, Victoria, BC, V8W 2Y2, Canada; Fisheries and Oceans Canada, 4160 Marine Drive, West Vancouver, BC, V7V 1N6, Canada; Fisheries and Oceans Canada, 4160 Marine Drive, West Vancouver, BC, V7V 1N6, Canada; Institut de Biologie Intégrative et des Systèmes (IBIS), Université Laval, Québec, QC, G1V 0A6, Canada; Institut de Biologie Intégrative et des Systèmes (IBIS), Université Laval, Québec, QC, G1V 0A6, Canada; Canada's Michael Smith Genome Sciences Centre, BC Cancer, Vancouver, BC, V5Z 4S6, Canada; Fisheries and Oceans Canada, 4160 Marine Drive, West Vancouver, BC, V7V 1N6, Canada; Fisheries and Oceans Canada, Pacific Biological Station, 3190 Hammond Bay Road, Nanaimo, BC, V9T 6N7, Canada; Fisheries and Oceans Canada, Pacific Biological Station, 3190 Hammond Bay Road, Nanaimo, BC, V9T 6N7, Canada; School of Aquatic and Fishery Sciences, University of Washington, Seattle, WA, 98105, USA; Facultad de Ciencias Veterinarias y Pecuarias, Universidad de Chile, Santa Rosa 11735, La Pintana, Santiago, 8820808, Chile; Millennium Nucleus of Austral Invasive Salmonids (INVASAL), Concepción, 4030000, Chile; Millennium Nucleus of Austral Invasive Salmonids (INVASAL), Concepción, 4030000, Chile; Facultad de Ciencias Agronómicas, Universidad de Chile, Santa Rosa 11315, La Pintana, Santiago, 8820808, Chile; Institut de Biologie Intégrative et des Systèmes (IBIS), Université Laval, Québec, QC, G1V 0A6, Canada; Department of Molecular Biology and Biochemistry, Simon Fraser University, Burnaby, BC, V5A 1S6, Canada; Department of Biology, Centre for Biomedical Research, University of Victoria, Victoria, BC, V8W 2Y2, Canada

**Keywords:** coho, silver salmon, Pacific salmon, genome assembly, SNPs, demographic history, genomics

## Abstract

Coho salmon (*Oncorhynchus kisutch*) are a culturally and economically important species that return from multiyear ocean migrations to spawn in rivers that flow to the Northern Pacific Ocean. Southern stocks of coho salmon in Canada and the United States have significantly declined over the past quarter century, and unfortunately, conservation efforts have not reversed this trend. To assist in stock management and conservation efforts, we generated a chromosome-level genome assembly. We also resequenced the genomes of 83 coho salmon across the North American range to identify nucleotide variants and understand the demographic histories of these salmon by modeling effective population size from genome-wide data. From demographic history modeling, we observed reductions in effective population sizes between 3,750 and 8,000 years ago for several northern sampling sites, which may correspond to bottleneck events during recolonization after glacial retreat.

## Introduction

**Fig. 1.**
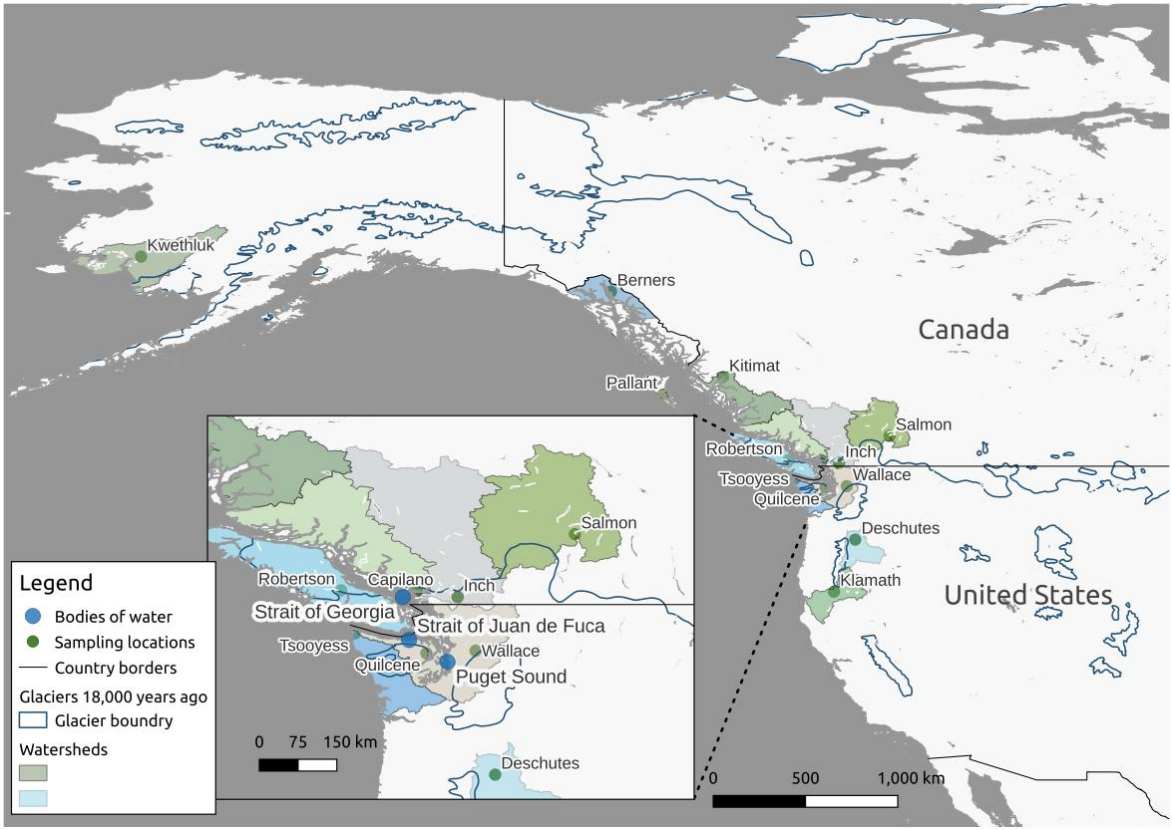
North Pacific Ocean with sampling locations and the extent of glaciers 18,000 years ago. This map was drawn in qGIS (QGIS Development Team 2022) with data from the following resources: watersheds (Pasos), glaciers (Government of Canada 2015), and all other information (Natural Earth). Watersheds were shown only if they contained a sampling location. The last glacial maximum has been estimated as ∼18,000 years ago [e.g. (Latch et al. 2009; Beatty and Provan 2010)]. The sampling locations for this study would be largely influenced by the Cordilleran Ice Sheet with an estimated maximum of ∼19,000–20,000 years ago (Clark et al. 2009), and if so, the glacial borders could be slightly conservative in this figure but still represent the extent of the North American ice sheets.

Coho salmon have special cultural significance to the people of the First Nations in British Columbia and have traditionally been one of the highest-value Pacific salmon in the commercial and recreational fishery sectors. In 1977, a climatic regime shift in the North Pacific Ocean ushered in 3 decades of increasing Pacific salmon production that culminated in 2009, when over 600 million salmon (1.1 million metric tonnes) were harvested (Irvine and Fukuwaka 2011). However, this increased production of salmon masked substantial variability in regional abundances and species composition. Whereas the productivity and harvest of chum (*Oncorhynchus keta*), pink (*O. gorbuscha*), and sockeye salmon (*O. nerka*) increased throughout the North Pacific after 1977, the opposite was true for coho and Chinook salmon (*O. tshawytscha*). These declines became particularly acute after 1989 when marine survival for these species began a downward spiral that has yet to be reversed (Beamish *et al*. 2010; Irvine and Fukuwaka 2011). A severe decline in the highly lucrative recreational coho salmon fishery in the Strait of Georgia saw the numbers of fish caught decrease from an average of over 500,000 to less than 100,000 throughout the 1990s (Beamish *et al*. 2011) (see Fig. 1 for a map of this region). In 2004, the recreational catch in the Strait of Georgia was just 9,500 coho salmon (Kristianson and Strongitharm 2006). Improved genetic resources and further studies may help us to better understand this decline and how to reverse it.

In a large-scale population structure analyses of coho salmon sampled from 318 localities, in 38 different regional groups in North America and Russia (representing most of the natural distribution of coho salmon), 17 microsatellite loci showed that salmon clustered geographically and regions could be delineated along a north—south gradient, with reduced variation to the north and isolated inland populations (Beacham *et al*. 2011). Follow-up studies, which used thousands of genetic markers in their analyses, observed that isolation-by-distance after the recolonization of northern streams from a main southern glacial refugium since the last glacial maximum could explain most of the patterns of genetic diversity in modern coho salmon across their North American distribution (Rougemont *et al*. 2020, 2022) [see Fig. 1 for the extent of North American ice sheets 18,000 years ago (Clark *et al*. 2009)].

After glaciers receded, previously inaccessible areas were once again habitable. It has been suggested that recolonization of these areas has influenced the present-day distribution of many other species, including plants [e.g. (Beatty and Provan 2010)] and other animals [e.g. (Latch *et al*. 2009)]. As noted in Beatty and Provan (2010), multiple North American refugia were possibly used by these organisms. For example, Arctic char (*Salvelinus alpinus*) are thought to have persisted in refugia north of the North American ice sheets (Moore *et al*. 2015) rather than in a southern refugium (as with coho salmon). With nearly 45% of the current freshwater habitat of Pacific salmon species under ice sheets during the last glacial maximum (Pitman *et al*. 2020), recolonization also played a major role in the modern distribution of coho salmon.

With genetic resources for conservation efforts in mind, and with the objective of expanding our understanding of recolonization after the last glacial maximum, the goals of this study were to: (1) generate a high-quality chromosome-level reference genome assembly and (2) estimate the timing of coho salmon recolonization of northern streams. Our method to construct a high-quality genome assembly was to use multiple sequencing technologies and techniques to complement each other. Our method to estimate the timing of recolonization was to resequence the genomes of 83 coho salmon and model effective population size from a comprehensive inventory of SNPs. This was done to identify genetic bottlenecks after the last glacial maximum corresponding to founding events during recolonization.

## Materials and methods

### Coho salmon samples for genome assembly

All animals were reared in compliance with the Canadian Council on Animal Care Guidelines, under permit from the Fisheries and Oceans Canada Pacific Region Animal Care Committee (under Ex.7.1). Using Inch Creek coho salmon (see Fig. 1 for location, NCBI BioSample accessions: SAMN05991263 and SAMN11962340), we generated fully homozygous diploid gynogenetic individuals [doubled haploids, see (Biagi *et al*. 2022) for full details] to help improve genome assembly quality. As noted in (Zhang *et al*. 2014), genome assemblies tend to benefit from the reduction of heterozygous genotypes, which might cause fragmentation during contig assembly.

Briefly, to produce doubled haploid individuals, a pressure shock (75.8 MPa for 5 minutes) was applied 6 hours post-fertilization (10°C) to coho salmon eggs that had been fertilized with UV-inactivated Atlantic salmon sperm (Chourrout 1984). Surviving individuals from experiments with Inch Creek coho salmon were grown until they could be non-lethally biopsied and their genotypes determined using a panel of 9 highly polymorphic microsatellite markers (Beacham *et al*. 2001) to assess homozygosity, non-paternity, and sex. Females that were homozygous at all loci and lacked any paternal alleles were retained and grown in freshwater for 8 months until they reached a size of 10–14 grams at which time selected fish were euthanized in 200 mg/L tricaine methanesulfonate (Syndel Laboratories, Canada) and sodium bicarbonate (400 mg/L). Tissue samples were stored at −80°C. Tissues from one coho salmon (NCBI BioSample SAMN05991263) were used for 10× Chromium, PacBio RS II and Sequel, and multiple Illumina sequencing libraries. Tissues from another individual (NCBI BioSample SAMN11962340) were used after the first coho salmon tissues were exhausted for Hi-C and further 10× Chromium sequencing.

DNA for Illumina sequencing was extracted from frozen muscle tissue using a Phenol:Chloroform extraction [adapted from (Sambrook *et al*. 2000)]; buffer ATL and Proteinase K (Qiagen) were used to digest tissue overnight. Phenol:chloroform:isoamyl alcohol (25:24:1) was added 50:50 to aqueous phase (3x) with a final extraction with chloroform:isoamyl alcohol (24:1). The aqueous phase was removed, and precipitated with 0.2 volumes of 10 M ammonium acetate and 2.5 volumes 95% EtOH. Precipitated DNA was extracted using a shepherd's crook and transferred to 70% EtOH to wash. DNA was resuspended overnight in buffer EB (Qiagen), and the DNA was quantified using a NanoDrop ND-1000 and a Qubit v2.0 Broad Range dsDNA kit (Life Technologies). The integrity of the DNA was confirmed by agarose gel electrophoresis.

High-molecular weight DNA was extracted from muscle tissue using a modified dialysis method. The tissue (550 mg) was ground into a powder with a mortar and pestle with liquid nitrogen. The powder was transferred to a 5 ml LoBind Eppendorf tube, along with 3600 ul buffer ATL (Qiagen), 400 ul proteinase K solution (Qiagen) and 40 ul RNAse A solution (Qiagen). The sample was digested at 56°C for 3 hours, with rotation at ∼4 rpm. A phenol-chlorform-isoamyl alcohol (25:24:1) purification was performed 3 times, followed by 1 round of chloroform-isoamyl alcohol (24:1). At each stage, 1 volume of organic was mixed with 1 volume of aqueous, inverted slowly for 3 minutes to mix thoroughly, spun for 15 minutes at 5000 g to separate the layers, and the aqueous top layer was transferred very slowly to a new tube using a 1000 ul pipette and wide bore tip. RNAse A solution (2 ul, 20 mg/ml Qiagen) was added and incubated at room temperature for 1 hr, followed by 5 ul Proteinase K (20 mg/ml) overnight at 4°C. Approximately 750 ul was obtained from each tube after the final stage. This volume was transferred to a Spectra/Por Float-A-Lyzer G2 1000 kD (pink) dialysis device, and dialysis was performed in 1 L of 10 mM Tris-Cl, pH 8.5 at 4°C with gentle mixing for 1 week, changing buffer 5 times. DNA quantity was determined by Qubit and quality by 0.6% agarose gel at 60 Volts.

Library preparation and sequencing details for the various sequencing technologies and samples can be found under the following NCBI submissions: 10× Chromium (SRX5975809, SRX5975837–SRX5975838), Hi-C (SRX5975810), PacBio Sequel (SRX5975835–SRX5975836), PacBio RS II (SRX2338326–SRX2338334), Illumina mate-pair (SRX2336577–SRX2336579, SRX2333332), and Illumina overlapping paired-end (SRX2333080—SRX2333081).

### Genome assembly

All PacBio data was assembled using Canu v1.8 (Koren *et al*. 2017). A genome size of 2.4 Gbp was estimated as input for Canu. Default settings were used with the exception of the following: “ovlMerThreshold = 2,000 corMhapSensitivity = normal correctedErrorRate = 0.085 minReadLength = 2,500 corOutCoverage = 200 ‘batOptions = -dg 3 -db 3 -dr 1 -ca 500 -cp 50’.”

Arrow v 2.2.2 from SMRTlink 6.0.0.47841 (PacBio) was used to polish the assembly with PacBio data with the ArrowGrid wrapper program (Koren *et al*. 2017). The assembly was also polished with Pilon v1.22 (Walker *et al*. 2014) using all the Illumina data generated for this project.

We performed scaffolding in 3 stages. In the first stage, BESST (Sahlin *et al*. 2014, 2016) was used to scaffold the assembly with the Illumina mate-pair data [prepared using NXTrim (O’Connell *et al*. 2015)]. Unknown and mate-pair fractions from NXTrim output were aligned to the polished genome with BWA mem 0.7.17 (Li 2014) and sorted with SAM tools 1.9 (Li *et al*. 2009). BESST was executed under default parameters, with data input in the order of (1) unknown and (2) mate-pair with inputs ordered smallest to largest target fragments size (all “-orientation rf”).

In the second scaffolding stage, the Tigmint—arcs—links pipeline was utilized. Tigmint v1.1.2 (default options) (Jackman *et al*. 2018) was run using the “arcs” pipeline to run all 3 stages. Within the Arcs v1.0.5 (Yeo *et al*. 2018) and LINKS v1.8.6 (Warren *et al*. 2015) portions of the pipeline, default parameters were used except a = 0.4 and l = 5 were used after parameter optimization.

A custom gap-closing script was run to attempt to fill newly scaffolded gaps with error-corrected PacBio data, for full details see (Christensen *et al*. 2020). Purge Haplotigs (Roach *et al*. 2018) was used to remove scaffolds with extremely low coverage. PacBio subreads were aligned to the genome assembly with Minimap2 (Li 2018), and a minimum coverage limit of 15 × was used to remove potential artifacts. A second round of Arrow polishing was performed as above. As gap sizes were no longer meaningful at this stage, and Tigmint introduced some very short fragments, sed (a Unix program) was used to remove contigs smaller than 200 bp nested within scaffolds and to resize all remaining gaps to 100 Ns. Bioawk fastx (https://github.com/lh3/bioawk) was used to remove any remaining scaffolds smaller than 1001 bp (<100 total removed).

After the Tigment pipeline, Hi-C data was aligned to the genome with Juicer 1.5.6 (Durand *et al*. 2016b). The parameters used were “-s Sau3AI and -S early” while also including Sau3AI cutsite file with the “−y” parameter. The resulting “merged_nodups.txt” file was input into 3d-dna v. 180,922 (Dudchenko *et al*. 2017) with “-i 50,000 -r 0.” A modified “.assembly” file, with ordered contigs/scaffolds, was created with data from a new genetic map generated from a 200 K Affymetrix SNP array (Barría *et al*. 2019). Scaffolds without mapped markers from the genetic map were identified and the order and orientation for these scaffolds were initially input into the assembly file based on synteny to the rainbow trout genome (Pearse *et al*. 2019) taken from the output of RaGOO (Alonge *et al*. 2019). Juicebox v1.8.8 (Durand *et al*. 2016a) was used to visualize and improve the assembly post-scaffolding. Purge_Haplotigs was used with the following settings—“contigcov -l15 -h 300 -m 155” and “purge -m 400” to allow for identification of “haplotigs” that represent high-percent identity, homeologous sequence.

Scaffolds that remained unlinked following manual review of the Hi-C data were repeat masked and aligned to the northern pike genome assembly (Rondeau *et al*. 2014) using LastZ (--nochain --gfextend --nogapped --identity = 75.0..100.00 --matchcount = 100 --format = general -hspthresh = 5,000). The northern pike was used due to its close relationship to salmonids and since it does not have a recent ancestral genome duplication, which is common to all salmonids (Leong *et al*. 2010). Scaffolds were ordered and oriented in the residually tetraploid-like regions of the genome to reflect the order in northern pike. The final genome was re-visualized with Hi-C in juicebox to remove any scaffolds for which Hi-C evidence did not support inclusion in at least one of the 2 tetraploid homeologues, then the Phase Genomics juicebox_assembly_converter.py script (https://github.com/phasegenomics/juicebox_scripts/; commit 7692ad5) was used to generate the NCBI AGP files. After manual editing to rename linkage groups based on prior assemblies and the northern pike linkage map (Rondeau *et al*. 2014) [identified through LastZ alignments in Geneious (Kearse *et al*. 2012)], the genome assembly was submitted to the NCBI. The assembly was uploaded to NCBI under the BioProject ID PRJNA352719. A Circos plot (Krzywinski *et al*. 2009) of the genome assembly was generated. Homeologous regions identified in this plot were detected using the pipeline from https://github.com/KrisChristensen/HomeologousRegionIdentification, and repetitive elements were detected using https://github.com/KrisChristensen/NCBIGenomeRepeats.

### Whole-genome resequencing and nucleotide variant calling

Whole-genome resequencing was used to identify SNPs across the coho salmon's North American range. Figure 1 and Table 1 identify sample locations (see Supplementary File S1 for more information). We included 1 commercial aquaculture strain from Chile as well for comparison (Table 1). Many of the samples are from hatchery sources. While this likely influences population level metrics, our main goal with these samples is to better understand the timing of recolonization of northern streams after the last glacial maximum. As most modern hatchery stock originates from local sources [e.g. (Heard 2012)], the influence of using hatchery samples is not expected to impact our interpretation of regional information. A more likely impact of sampling from hatcheries is that modern effective population size may differ from wild populations and we consider this in our interpretations of the data.

**Table 1.** Whole-genome resequencing sampling locations.

Source	Country	State/province	Count female, male
Klamath River (Hatchery)	USA	CA/OR	1F, 4M
Deschutes River (Hatchery)	USA	CA/OR	2F, 3M
Big Quilcene River (Hatchery)	USA	WA	2F, 3M
Wallace River (Hatchery)	USA	WA	6 M, 4?
Tsoo-Yess River (Hatchery)	USA	WA	1F, 4M
Inch Creek (Hatchery)	Canada	BC	3F, 5M
Capilano River (Hatchery)	Canada	BC	5F
Robertson Creek (Hatchery)	Canada	BC	5M
Salmon River (Hatchery)	Canada	BC	5F
Pallant Creek	Canada	BC	5M
Kitimat River (Hatchery)	Canada	BC	5F, 5M
Berners River	USA	AK	2F, 3M
Kwethluk River	USA	AK	1F, 4M
AquaChile (Strain)	Chile	NA	5F

DNA for resequencing was extracted from fin-clips using the DNeasy Blood and Tissue extraction kit (Qiagen) or a MagMAX DNA Multi-Sample Ultra Kit with a KingFisher (ThermoFisher Scientific). Following DNA extraction, samples were quantified by Qubit BR DNA assay (ThermoFisher) and integrity validated by agarose gel electrophoresis. At McGill University and Genome Québec Innovation Centre (Montreal, QC, Canada), individual Illumina libraries were constructed with Illumina TruSeq LT sample preparation kits, and each individual was sequenced either on an Illumina HiSeq2500 (PE125) or a HiSeqXTen (PE150), targeting approximately 15–30× coverage. Resequenced genomes were submitted to the NCBI under BioProjects: PRJNA401427 and PRJNA808051 (Supplementary File S1).

BWA-MEM v0.7.17 (Li 2013) was used to align Illumina data to the reference genome, with the -M option for Picard compatibility. The Picard v2.18.9 (github.com/broadinstitute/picard) AddOrReplaceReadGroups program was used to add read group IDs, and the MarkDuplicates program was used to mark duplicates (default settings). GATK v3.8 (McKenna *et al*. 2010; Van der Auwera *et al*. 2013) was then used to call genotypes. Base and variant recalibration were each performed once (for 2 rounds through genotyper). The variants used for recalibration were from 1) a reduced set of very high-confidence calls following default “hard-filtering” guidelines from GATK documentation from the first round of genotyping with a particular focus on coding regions and 2) validated SNPs from a 200 K Affymetrix SNParray [validated in a previous study (Barría *et al*. 2019)].

Following genotyping, VCFtools v0.1.15 (Danecek *et al*. 2011) was used to additionally filter data to only include biallelic SNPs with a minor allele frequency of 0.05 or greater, variants with fewer than 10% missing genotypes, and variants with a mean coverage between 5 and 200. Finally, the SNPs were filtered for linkage disequilibrium to reduce the influence of large haploblocks [bcftools (Danecek *et al*. 2021) version 1.9–102-g958180e + prune -w 20 kb -l 0.4 -n 2]. See Table 2 for SNP filtering parameters used for each of the analyses in the following paragraphs.

**Table 2.** SNP filtering for different analyses.

Parameters	Bi-allelic, < 10% missing, coverage 5–200	^a^MAF 0.05	^a^Linkage disequilibrium
Analysis(es)	SMC++	Pi, Fis, observed heterozygosity, private allele count	PCA, Mantel test
Number of variants	14,397,038	5,631,459	152,128
Average SNP coverage	21.6x	21.9x	21.6x
Average missing genotypes	17,665	11,780	514

Represents additional filters to the previous category.

### Basic population metrics and demographic history modeling

A principal components analysis (PCA) was performed using PLINK (Chang *et al*. 2015) v1.90b6.15 with default parameters (https://www.cog-genomics.org/plink/1.9/). Private allele counts per river (river is used instead of sampling site throughout the rest of the text) were tallied using the populations module in Stacks (Catchen *et al*. 2011, 2013) version 2.54 with default parameters. Populations with more than 5 individuals were randomly subsampled to 5 to reduce the influence of uneven sampling on the number of private alleles identified. Stacks was also used to calculate other population level metrics such as observed heterozygosity, nucleotide diversity (Pi), and Fis with default settings. A Mantel test was performed in R (R Core Team 2020) with the following packages to test isolation-by-distance: adegenet (version 2.1.7) (Jombart 2008; Jombart and Ahmed 2011), ade4 (Dray and Dufour 2007; Bougeard and Dray 2018; Thioulouse *et al*. 2018), and vcfR (version 1.13.0) (Knaus and Grünwald 2017).

To infer demographic histories of the salmon from the various rivers, we used SMC++ (Terhorst *et al*. 2017) version 1.15.4.dev18 + gca077da. In this analysis, we set the mutation rate to 8e–9 bp/generation and the generation time to 3 years. These parameters were previously used in another coho salmon study examining demographic histories (Rougemont *et al*. 2020). We used nucleotide variants that were not filtered for rare variants (e.g. MAF < 0.05). We also used the –missing-cutoff option (50 kbp) in SMC++ to reduce the influence of missing genotypes (e.g. in centromeres). While each study site was sparsely sampled (Table 1), for this analysis we were mainly interested in understanding bottleneck events that occurred thousands of years ago. These events should be echoed in the genomes of a majority of individuals from a particular location as most presumably originated from the same founding event (geographically and chronological). Salmon tend to return to the same body of water or a suitable nearby location (Quinn 1993). For recolonization, we would expect major bottleneck events after the last glacial maximum in all of the streams covered by ice-sheets.

## Results

### Genome assembly

The size of the coho salmon genome assembly was 2.3 Gb, which is consistent with that for the closely related Chinook salmon genome assembly (NCBI accession: GCF_018296145.1). The contig N50 of the assembly was 1,159 kb and the percent of complete BUSCOs was 99%, which is comparable to the human genome assembly also at 99% (NCBI accession: GCF_000001405.39) (Table 2).

**Fig. 2.**
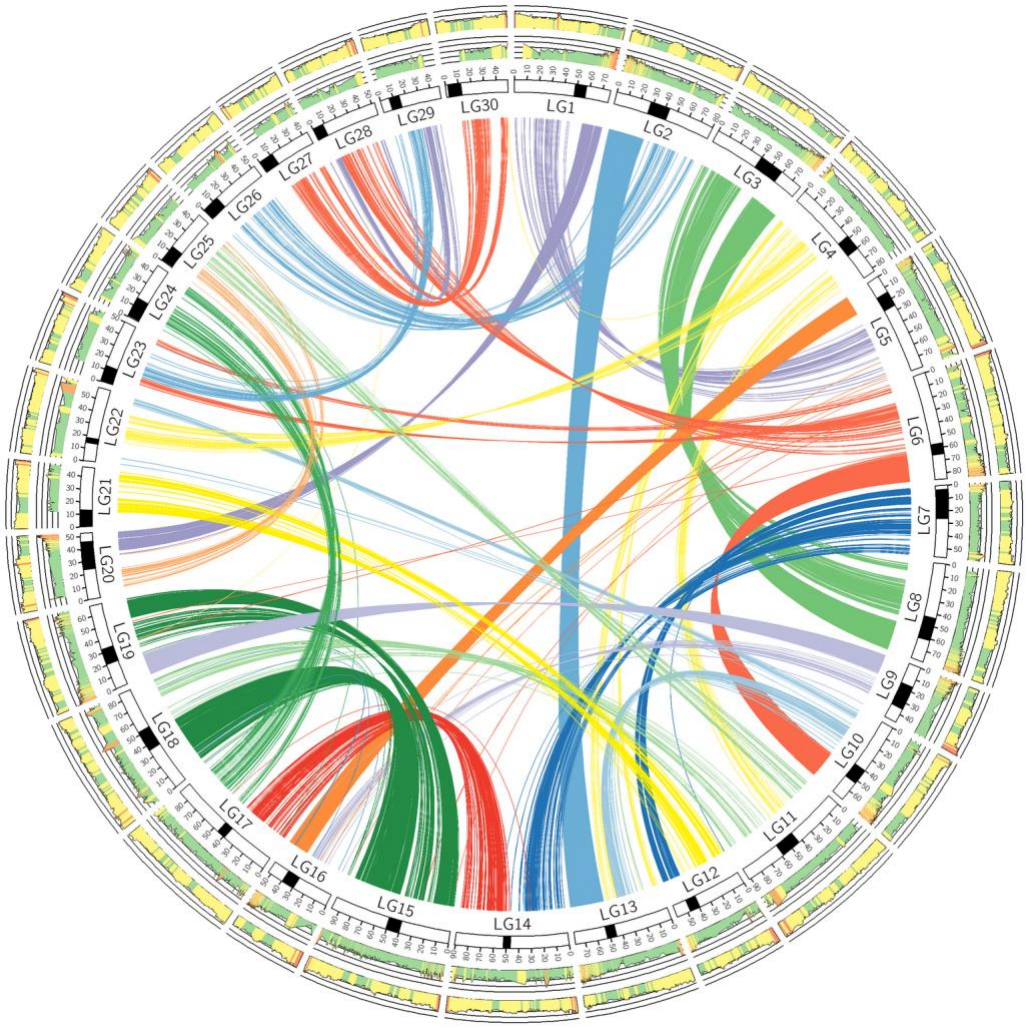
Circos plot of the coho salmon genome assembly. In the interior of the Circos plot, the links between duplicated regions of the chromosomes/linkage groups (i.e. homeologous regions) are shown. The ring outside of these links contains representations of the chromosomes with the approximate position of the centromere marked by a filled region [centromere positions from (Kodama et al. 2014)]. The tick marks represent 10 Mbp intervals. The next ring contains the percent identity between duplicated regions of the chromosome. The red color represents very high similarity (> 98%), the orange color high similarity (94–98%), the yellow moderate (91–94%), and the green low (< 91%). On the outer ring, the fraction of repetitive elements is shown, with red representing high (> 70%), yellow as moderate (45–70%), and green as low (< 45%).

The coho salmon genome has extensive signatures of chromosomal duplication (Fig. 2), which have been retained from the whole genome duplication common to all salmonids (Allendorf and Thorgaard 1984). The majority of homeologous regions from the salmonid-specific genome duplication have diverged by at least 9% (*i.e.* ≤ 91% identity, Fig. 2), but certain sections of the genome have retained high sequence similarity (Fig. 2). Regions with very high sequence similarity remained as unplaced scaffolds (likely collapsed into 1 sequence) as it was not possible to resolve which sequence belonged to which duplicated region (see assembly methods available on the NCBI website, accession: GCF_002021735.2).

The coho salmon genome also has a high retention of repetitive elements (Fig. 2; Table 3), which is another commonality of studied salmonids [e.g. (Lien *et al*. 2016; Christensen *et al*. 2020)]. This is especially true in regions near the centromere where the fraction of repetitive elements is roughly 60–70% (Fig. 2). That value is high compared to the genome average of 53% (Table 3). For comparison, the most recent version of the Chinook salmon genome also has a repeat content of 53% (NCBI accession: GCF_018296145.1).

**Table 3.** Genome statistics.

Contig N50	Contig #	BUSCO	% Repeats	Genes
1,159,298	8,770	99.2%–57.1:42.2^a,b^	53.12^b^	60,330^b^

Percent complete-single:duplicate.

Reported by NCBI (NCBI used actinopterygii_odb10 for BUSCO).

NCBI: GCF_002021735.2.

### Basic population metrics and demographic history modeling

**Fig. 3.**
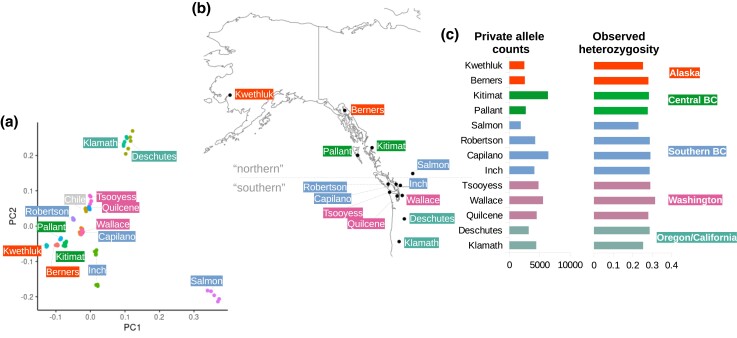
Summary statistics of coho salmon sampling sites. a) A PCA of coho salmon individuals [plotted using ggplot (Wickham 2016)]. Individuals from the same sampling site have the same color. b) A map of the various river sampling sites of this study [plotted with the maps (Becker et al. 2018) package in R]. c) Private allele counts and observed heterozygosity values are also displayed to the side as bar graphs.

A PCA of 83 resequenced coho salmon genomes sampled from across North America (see Table 2 for the number of SNPs for analyses), revealed that coho salmon clustered by region with the exception of the Salmon River (Fig. 3). This river belongs to the Thompson River watershed, and coho salmon from this region have previously been observed to cluster in a similar manner (Rougemont *et al*. 2020). Clustering otherwise appeared to be dependent on latitude. However, isolation-by-distance was not detected (*P*-value = 0.068). In this study, we had fewer sampling sites and fewer samples in general than previous studies that did find a significant correlation of genetic distance and geographic distance (Rougemont *et al*. 2020, 2022).

The Salmon River group had the lowest private allele count (1,876 vs. a median of 4,188) and observed heterozygosity (0.22966 vs. a median of 0.285565, Fig. 3). The regions with the highest private allele count appears to be around the Puget Sound (e.g. Wallace River, private allele count = 5,546) and the Strait of Georgia (e.g. Capilano River, private allele count = 6,415). Most of the northern rivers have low private allele counts with the exception of the Kitimat River (private allele count = 6,341), which has the second highest count (Fig. 3).

**Fig. 4.**
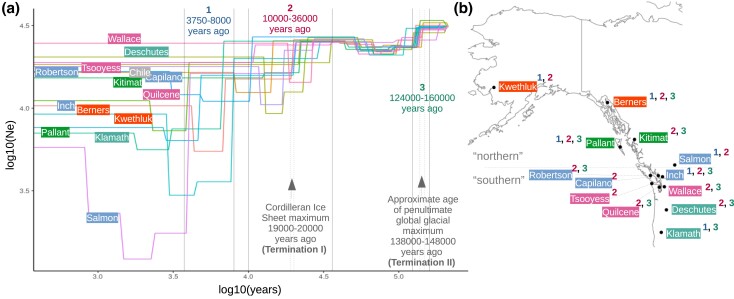
Demographic histories of coho salmon based on genome resequencing. a) Each labeled line represents multiple individuals from the same river or strain. The X-axis represents calendar years based on a generation time of 3 years for coho salmon. The Y-axis is the effective population size (Ne) estimate. The estimated age of the Cordilleran ice sheet maximum was taken from (Clark et al. 2009). The approximate age of the last interglacial period was based on (Chappellaz et al. 1990). b) For each location, a number nearby indicates a drop of at least 5,000 in the Ne for one of the time intervals noted in A.

To put the nucleotide variation generated by isolation-by-distance [identified previously (Rougemont *et al*. 2020, 2022), but not in the current study] into a broader context, we identified possible times when northern populations could have recolonized after the last glaciation period. By modeling demographic histories from resequenced genomes with the SMC++ program, we were able to identify major decreases in effective population size (Ne) that correspond with the Cordilleran Ice Sheet maximum and the presumed penultimate global glacial maximum (Fig. 4). We also observed that for some sampling sites, mostly northern, there was an additional drop in effective population size between 3,750 and 8,000 years ago (Fig. 4).

## Discussion

The BUSCO metric of this genome assembly suggests that it contains a near complete representation of the gene content of this species, and is comparable to model species. The other metrics demonstrate that this genome assembly is contiguous and suitable for downstream analyses. We performed several analyses on the genome assembly itself to identify homeologous regions and repetitive elements. We then measured several basic population metrics and modeled demographic histories from resequenced genomes.

As with previous analyses of salmonid genomes (Allendorf and Thorgaard 1984; Berthelot *et al*. 2014; Lien *et al*. 2016; Christensen *et al*. 2018, 2020; De-Kayne *et al*. 2020), the retention of duplicated chromosomes from the salmonid-specific whole genome duplication (Allendorf and Thorgaard 1984) and the retention of repetitive elements are defining features of the coho salmon genome. Some of the duplicated regions have likely retained very high sequence similarity for roughly 90 million years [time estimate from (Allendorf and Thorgaard 1984; Berthelot *et al*. 2014; Macqueen and Johnston 2014)]. A possible mechanism for high sequence similarity retention is through tetrasomic inheritance (Allendorf *et al*. 2015).

After completing the high-quality genome assembly, we measured population metrics from 83 resequenced genomes. One of the striking features of the PCA of coho salmon populations was how divergent Salmon River salmon were to all other populations. The Salmon River is part of the Thompson River watershed and coho salmon from this system were thought to be isolated from all other populations for potentially 150,000 years before secondary contact roughly 13,500 years ago (essentially during the previous glacial period) (Rougemont *et al*. 2020). This would be consistent with findings in kokanee (*O. nerka*, a landlocked sockeye salmon ecotype) in the upper Columbia River that similarly appear divergent from all other populations of sockeye salmon and kokanee (Christensen *et al*. 2020). Taken together, these pieces of evidence might provide support for a glacial refugium near the intersection of the Cordilleran Ice Sheet and the Laurentide Ice Sheet.

It is also possible that another unknown factor was influencing past analyses and the PCA from the current study. The Salmon River coho salmon have reduced heterozygosity and reduced private alleles, which are indicators of a recent and extensive bottleneck. We were also able to infer the demographic history from whole genome sequences of the Salmon River coho salmon and found evidence of a bottleneck (from ∼Ne 16,227 to ∼Ne 1,749) occurring in this population around 4,000 years ago. We only collected samples from one tributary of the Thompson River (a part of a much larger basin) and can only suggest that a plausible hypothesis from this data is that recolonization of the Salmon River from a small founding population took place after glaciers receded. We did not account for the influence of hatcheries, which could also influence some of the metrics discussed above. Also, we did not incorporate linked selection in demographic modeling as the type of analysis that we used was not amenable. Without linked selection accounted for, there could be biases in times and effective population sizes from our estimates (Pouyet *et al*. 2018).

Other streams clustered in the PCA based largely on latitude for both PC1 and PC2 of the PCA. With a much more extensive sampling strategy, Rougemont *et al*. (2020) found a similar trend and tested various demographic histories. The authors of that study found that the best supported model was a glacial refugium to the south with recolonization of the northern streams after glacial retreat—generating genomic signatures of isolation-by-distance. In our study we did not detect significant isolation-by-distance, but we had fewer sampling sites and samples in general. The private allele analysis identified that most of the northern streams did have low private allele counts compared to southern streams, which would be consistent with the hypothesis suggested by Rougemont *et al*. (2020).

From inferred demographic histories, we were able to estimate a recolonization date of some northern streams (based on the founder effect that would be expected to accompany recolonization) to between 3,750–8,000 years ago. These values are based on assumptions of a mutation rate of 8e–9 bp/generation and a generation time of 3 years. Linked selection may also bias our time and effective population estimates (Pouyet *et al*. 2018) as we did not account for them in modeling. However, these recolonization estimates are in-line with recolonization estimates of 4,000–12,000 based on continental glaciation and geographic data proposed previously (Beechie *et al*. 2001; Waples *et al*. 2008).

While it is important to remember that time and population estimates are influenced by many factors when inferring demographic histories from sequence data, multiple lines of evidence can be used to strengthen these inferences or put them in a more realistic context. Radiometric evidence supports that the Cordilleran Ice Sheet maximum occurred between 19,000 and 20,000 years ago (Clark *et al*. 2009). Likewise, chemical properties of gases in Antarctic ice cores support the termination of the last glaciation period (Termination I) to roughly the same time period, as well as a previous termination of the penultimate glaciation period around 138,000 and 148,000 years ago (Termination II) (Kawamura *et al*. 2007). In the demographic histories of the coho salmon, we noted dramatic declines of nearly all salmon populations for both these time periods. This observation supports the parameters used for modeling the demographic histories as we expect that populations might decline in response to increased glaciation or rapid climate change.

The overall trend we observed from modeling demographic histories was major drops in effective population size at each transition from glaciation to inter-glaciation period with increases for nearly all populations after the penultimate glaciation period and uncommon increases for specific rivers after the most recent glaciation period. At a species level, these transitional drops in effective population size likely influence multiple aspects of coho salmon biology since genetic variability can contribute to many characteristics of a species.

## Conclusions

In this study, we generated a high-quality reference genome assembly as a tool for conservation and management of coho salmon. Additionally, we resequenced the genomes of a wide distribution of coho salmon from rivers along North America to identify nucleotide variants that will have significant utility for other analyses of coho salmon genetics. We modeled demographic histories of the coho salmon with this data and estimated recolonization occurred between 3,750–8,000 years ago for some northern streams, and observed that major reductions in effective population size were related to changes between glacial and inter-glacial periods. This coho salmon genome assembly will facilitate research to better understand coho salmon biology in the future and will enhance management of this culturally and economically important species.

## Supplementary Material

jkad033_Supplementary_Data

## Data Availability

Raw data for the genome assembly was submitted to the NCBI under the BioProject PRJNA352719. Whole genome resequencing data was submitted under PRJNA401427 and PRJNA808051 to the NCBI BioProjects (see Supplementary File S1 for specific samples used in this study). The VCF file used for analyses in this study is available at figshare: https://doi.org/10.25387/g3.20032082. Supplemental material available at G3 online.
