# Supplementary material for: Population-size history inferences from the coho salmon (Oncorhynchus kisutch) genome
Source: G3 (Bethesda). 2023 Feb 10;13(4):jkad033. doi: 10.1093/g3journal/jkad033 (PMC10085799; doi:10.1093/g3journal/jkad033)
Supplement: jkad033_Supplementary_Data [file jkad033_supplementary_data.zip › Supplemental_Material_Legends_G3-2022-403948.docx]

**Supplemental Material**

**File S1. Sample information, kinship information, and SRA accession numbers.**
